# One year of Communications Engineering

**DOI:** 10.1038/s44172-023-00078-z

**Published:** 2023-05-26

## Abstract

Today we celebrate the first anniversary of the launch of *Communications Engineering*. We reflect on our progress and welcome in the second year by announcing some new projects and content.

The first year of *Communications Engineering* has been one of growth and learning for our editorial team. At the time of writing, we have published 54 research papers covering a breadth of topics across all the major engineering disciplines. All our research publications have been editorially selected to offer new thinking with impact for a specialist engineering community and have gone through careful peer review managed by our editorial team. We have published a variety of Comment and Analysis pieces including Research Highlights, Q&A and Viewpoints which we hope will engage and inspire our growing engineering audience. We are delighted that a number of our papers have received wider media coverage, the result of press releases from either the relevant institutional or the Nature group Press Office. Some of our research papers have been covered in Highlight Articles from various *Nature* journals, and some have received multimedia attention.

## Bringing communities together

In our launch editorial^1^ we described three ways in which we aim to bring the engineering research community together. First we want to create a sense of a shared mission in addressing the Sustainable Development Goals. We have released Calls for Papers on the topics of Battery Management Systems, Energy Infrastructure (in collaboration with *Nature Communications*) and Thermal Engineering for Sustainability. And today we launch a new Call for Papers on the topic of engineering advances to tackle solid waste streams, such as food, paper and textiles, electronics, plastics and packaging, and municipal solid waste. Second we want to encourage and support engineering fields less familiar with the Nature Portfolio. We published a Special Issue combined with a Call for Papers on the broad topic of Resilient Infrastructure, welcoming submissions in civil and transport engineering amongst other topics. We have also launched a Call for Papers on the theme of Deployable and Reconfigurable Structures, a topic of high interest and relevance to the mechanical and structural engineering communities. Third, we want to support interdisciplinarity. Today we release a new retrospective and rolling collection on a topic in which we have published a number of interesting contributions: Bio-inspired Engineering. Papers in the collection come from the fields of aerospace, imaging, robotics and materials design. In support of this collection we publish today a review from the group of Aimy Wissa on the topic of bio-inspired flow dynamics^2^. All of our Calls for Papers are still live at the time of writing, and welcoming submissions (https://www.nature.com/commseng/collections).

Today we bring communities together in another way, by publishing the first in a short series of Q&As exploring the connectivity between engineering academia and industry. Our editorial board member Inge Herrmann offers her insights on the challenges and opportunities offered by commercialisation of academic research particularly in the field of biomedical engineering^3^. We hope that this will be particularly stimulating for early career researchers interested in entrepreneurship. Do keep a look out in future months for Q&As exploring more aspects of this interface. Our recently launched Industry Showcase also highlights all our research content from industrial or academic/industrial collaborations^4^; future industry publications will be added to this Showcase.

## Our service

We are grateful to all the researchers who have placed their trust in us with their research contributions. One key goal of the journal is to provide a high quality author service. As we await the official publication of our first set of peer review metrics, we continue to work hard with our editorial board, exploring new processes and strategies to optimise turnaround times for our authors. There are inevitably going to be delays with some submissions, and we aim to keep our authors informed of the progress of their submission to minimise the pain during the wait while their paper is being assessed.

We are also grateful to our peer reviewers for contributing their time and effort in assessing our submissions. Their hard work can often be seen in the peer review files supporting those papers for whom the authors have selected transparent peer review. In a further effort to recognise our peer reviewers, today we begin our Reviewer of the Month Programme. Each month we will recognise one reviewer whom we feel has made a significant effort to provide careful analysis and thoughtful insight and advice to help researchers improve their publications. We are delighted to announce that Megan O. Hill, Herchel Smith Postdoctoral Fellow from the University of Cambridge will be our first Reviewer of the Month.

## Improving representation

Our final goal mentioned in our launch editorial was to support historically under-represented groups, with a key first effort being to actively include and represent women in our pages and our teams. At the time of writing, our editorial board has a balance of 50:50 representation of males and females. In addition, all our guest edited collections have at least one female guest editor. In another effort towards female representation, in June 2022, we supported International Women in Engineering Day (INWED) by publishing a Viewpoint where seven of our editorial board members wrote a short piece recognising a woman researcher who has been influential in their academic career^5^. Next month is our second INWED, and we will outline in more detail then how our journal continues to support women in the engineering community.

## From the ground up

Over the past year we have been working hard to build the foundations of an engaging and respected broad scope engineering journal. We invite you to explore our content, to dig into research and discussion on topics within and beyond your own specific area of research study. We hope you will find something new and interesting and that you will come back and visit our journal again soon for more insight and innovation from across the engineering research community.
